# Thromboembolic Risks in Pancreatic Ductal Adenocarcinoma: A Review of Anatomical and Clinical Variations

**DOI:** 10.1155/cjgh/7566020

**Published:** 2026-03-23

**Authors:** Lei Zhang, Weili Zheng, Yi Bao

**Affiliations:** ^1^ Department of Oncology, The Second Affiliated Hospital of Jiaxing University, Jiaxing, 314000, Zhejiang, China, zjxu.edu.cn

**Keywords:** pancreatic ductal adenocarcinoma (PDAC), radical pancreatic surgery, secondary thrombocythemia, splenectomy, venous thromboembolism (VTE)

## Abstract

This review article presents the anatomical and clinical variations in patients with pancreatic ductal adenocarcinoma (PDAC) arising from the head and body/tail regions. It discusses the distinctive risk factors for venous thromboembolism (VTE) formation and assesses various parameters for monitoring VTE and intervention strategies. Pancreaticoduodenectomy is typically performed for tumors in the head region of the pancreas, while distal pancreatectomy with splenectomy is performed for tumors arising from the body or tail of the pancreas. This article also reviews the stratified analysis of VTE risks in PDAC based on tumor origin, aiming to establish valuable VTE risk factor assessment tools and prophylactic anticoagulation strategies. Furthermore, it focuses on PDAC patients with tumors arising from distinct pancreatic anatomical locations to improve quality of life, reduce thrombotic risks, and optimize clinical outcomes.

## 1. Introduction

Pancreatic cancer (PC) ranks as the seventh leading cause of all cancer‐related deaths worldwide and the sixth in China [1, 2]. Although the improvement of novel anticancer therapies includes targeted therapies and immunotherapies, the 5‐year survival rate of PC is still no more than 13% [3]. Only 10%–15% of patients with PC are eligible for surgical resection, as patients with PC manifest their symptoms only at advanced stages [4].

Pancreatic ductal adenocarcinoma (PDAC) is the most common pathological subtype of PC, accounting for more than 90% of diagnoses [4]. According to its anatomical location, PDAC is further categorized into head and body/tail origins. Head PDAC accounts for about 75% of cases, while body/tail PDAC makes up the remaining 25% [5]. Distinctive risk factors influence thromboembolism formation between the head versus the body/tailPDAC patients due to variations in cellular composition, blood supply and innervation, and differences in surgical procedures.

The formation of venous thromboembolism (VTE), specifically portal vein thrombosis (PVT), frequently occurs in PDAC patients who are normally in a hypercoagulable state. In a meta‐analysis studying the risk of VTE in 18 cancer types, patients with PC were shown to have the highest 1‐year risk of VTE among patients of mixed demographics, disease stage, and treatment characteristics [6]. In another prospective, observational study, VTE appeared to be a frequent and early‐onset complication in patients diagnosed with PDAC (approximately 20%) and was associated with significant decreases in times of PFS and OS [7]. However, studies from various countries have reported different prevalence rates of VTE in patients with PDAC [8–10], and relatively low incidence of VTE in Asia (approximately 11%) followed by a meta‐analysis from 8972 PDAC patients from 24 clinical studies [11]. Nevertheless, VTE is associated with an unfavorable prognosis in PDAC patients [12]. Therefore, understanding the epidemiology and risk factors of thromboembolism in PDAC is crucial for decreasing morbidity and mortality rates.

This article aims to review the distinctive risk factors contributing to VTE formation in PDAC patients with anatomical and clinical variations associated with tumors arising from head and body/tail of the pancreas to provide valuable insights for future clinical trials on VTE prevention and intervention strategies.

## 2. Methods and Search Strategies

A PubMed search of English‐language articles described PDAC including clinical studies (randomized clinical trials, prospective and retrospective observational studies), meta‐analyses, and systematic reviews. The following search words were selected: “pancreatic cancer”; “pancreatic ductal adenocarcinoma OR PDAC”; “head pancreatic ductal adenocarcinoma OR head PDAC”; “body/tail pancreatic ductal adenocarcinoma OR body/tail PDAC”; “venous thromboembolism OR VTE”; “deep vein thrombosis OR DVT”; “cancer‐associated thrombosis”; and “prophylactic anticoagulation in cancer‐associated thrombosis”.. Priority was given to the selection of high‐quality articles that weSre published between January 1, 2010, and June 1, 2025.

## 3. Anatomical and Clinical Variations in Patients With PDAC Originating From the Head or Body/Tail

The patients with PDAC are categorized into head and body/tail origins due to anatomical location, variations in cellular composition, blood supply, innervation, and surgical intervention procedures. By the sixth week of the development of the human embryo, the common duct and ventral pancreatic bud rotate 180° clockwise around the duodenum [13]. The ventral bud, located under the dorsal bud, forms the head’s inferior part and the uncinate process of the pancreas, each with its own duct [14]. By the seventh week of gestation, the buds fuse, and part of the dorsal bud duct atrophies occurring in most individuals, while the remaining ducts form the pancreatic duct connecting to the duodenum via the major papilla [14]. The lymphatic drainage of the pancreas is a crucial aspect of its anatomy and is primarily responsible for draining lymph from the organ to regional lymph nodes. The lymphatic vessels from the head of the pancreas typically follow the arterial supply, entering the pancreaticoduodenal nodes before draining into the celiac lymph nodes [15]. Lymphatic vessels from the body and tail regions of the pancreas primarily drain into the splenic lymph nodes and the inferior pancreaticoduodenal lymph nodes [15]. The difference in lymph node drainage can significantly influence prognosis and treatment strategies in patients with PDAC. The genetic landscape of PDAC has been found to profoundly affect tumor behavior, which may influence tumor progression. KRAS mutations, a hallmark feature occurring in approximately 85%–90% of cases, have been intensively investigated for their roles in various aspects of tumor biology, including proliferation, survival, and metastasis [16]. In a study of PDAC patients from East Asia, KRAS mutations were reported in 97.1% of body/tail PDACs compared to 82.4% in head PDAC patients [17]. In another study from the USA, it was found that KRAS mutations were present in 93.8% of body/tail tumors compared to 90.2% in head tumors; however, the difference did not reach statistical significance [18]. Tumors harboring mutated KRAS genes are positively associated with worse survival outcomes for PDAC patients whether or not they undergo surgical resection [19]. For subsequent studies of KRAS subtype mutations, the G12D mutation is more frequently observed in head PDAC patients, while the G12V mutation is predominantly found in body/tail tumors [20].

Due to anatomical and clinical variations between head and body/tail PDAC, the surgical resection approaches for PDAC patients are different. Pancreaticoduodenectomy is a standardized surgical procedure for patients with PDAC located in the head of the pancreas [21]. Conversely, distal pancreatectomy with splenectomy is the preferred surgical procedure for PDAC patients originating in the body/tail region [21]. To our knowledge, the effect of tumor location within PDAC on the incidence of VTE after surgical resection has not been systematically investigated. This relationship remains to be further clarified. Only a few studies have suggested differences in the risk of VTE based on tumor site in PDAC. For instance, a meta‐analysis showed that patients whose tumors were located in the body and tail of the pancreas had a higher risk of VTE than those with tumors in the head of the pancreas [11]. In fact, many studies on the incidence and risk factors for the occurrence of VTE in PDAC from the head and body/tail do not differentiate between them and often treat them as a single tumor type. However, the diverse surgical approaches for resectable PDAC result in different postoperative complications. For instance, secondary thrombocythemia after splenectomy in tumors located in the body/tail region may give rise to the risk of developing VTE. Therefore, stratifying risk factors in studies of clinical outcomes of PC originating from the head or body/tail regions is important for evaluating the prophylactic management of VTE. Table 1 summarizes the differences in embryonic origin, genetic features of KRAS mutations, surgical procedures, etc., between PDAC derived from the head and body/tail regions.

**TABLE 1:** A summary table comparing head vs. body/tail PDAC.

Characteristics	Head PDAC	Body/tail PDAC
Embryonic origin	Ventral pancreatic bud (with the rotation of the common bile duct)	Dorsal pancreatic bud (main part)

Symptoms	Easily cause biliary obstruction (jaundice) and duodenal obstruction	Many patients are asymptomatic in early, easily invading nerves and splenic vessels

Lymphatic drainage	Enter the pancreaticoduodenal nodes before draining into the celiac lymph nodes	Primarily drain into the splenic lymph nodes and the inferior pancreaticoduodenal lymph nodes

Genetic features of KRAS mutations	Reported 82.4% in East Asia [17]	Reported 97.1% in East Asia [17]
	Reported 82.4% in USA [18]	Reported 82.4% in USA [18]

Surgical procedure	Pancreaticoduodenectomy	Distal pancreatectomy with splenectomy

## 4. Assessment of Different Risk Parameters for Thromboembolism in Patients with PDAC

Patients with malignancies often experience cancer‐associated thromboembolism due to a hypercoagulable state, which in turn exacerbates cancer‐related mortality. Studies have shown that elevated P‐selectin and thrombin in PDAC can mediate the adhesion of platelets to PC cells [22, 23]. High plasma levels of soluble P‐selectin (sP‐selectin) are suggested to be a biomarker in those patients with acute VTE both in cancer patients and without cancer [24, 25]. The hypercoagulable state further exacerbates the overall risk of thromboembolism due to surgical interventions, decreased physical activity, and adjuvant chemotherapy in patients with PDAC. To prevent the occurrence of VTE and to enable earlier assessment of VTE in PDAC patients, numerous studies have been conducted to validate the efficacy of these scales using established VTE risk‐factor assessment tools.

Currently, the Caprini score is used as a preoperative assessment tool for evaluating VTE and bleeding events in surgical patients [26, 27] (Table 2). According to the 2023 European Society of Oncology (ESMO) guidelines, a Caprini score ≥ 5 is an indicator of high risk for VTE in patients with malignancies [28]. The Padua Prediction Score (PPS) is another VTE risk‐factor assessment tool developed to help clinicians identify hospitalized patients at risk for VTE and to determine the need for prophylactic anticoagulant therapy (Table 3) [29]. The Khorana Score (KS) is designed for different cancer types aiming to improve early detection or prophylaxis in these malignant patients with a high risk of VTE occurrence (Table 4) [30]. The PROTECHT score is a modification of the KS that adds platinum or gemcitabine—two chemotherapy drugs frequently used in patients with PDAC—assigning one point each (Table 5) [31]. Indeed, refined risk assessment tools stratifying PDAC patients with distinct origins using additional new biomarkers are required to provide valuable strategies for prophylactic anticoagulant interventions in high‐risk patients. For instance, BMI ranging from 30 to 35 kg/m^2^ has been included as a risk parameter taken into account for VTE risk points. However, in real‐world scenarios, severe obesity is extremely rare in patients with PC. The value of utilizing BMI ≥ 30–35 kg/m^2^ as a predictive factor in KS or PROTECHT score for PDAC patients remains controversial. It is worth noting that Type II diabetes mellitus, a disease defined by persistent hyperglycemia, is frequently associated with PC patients, and subsequently hyperglycemia is also deemed to be a significant risk factor for atherosclerosis and thrombosis [32]. Hyperglycemia is caused by impaired insulin sensitivity and an inadequate compensatory insulin response in Type 2 diabetes mellitus. Recently, the term Type 3c diabetes has been defined to describe diabetes secondary to chronic pancreatitis, PDAC, cystic fibrosis, and previous pancreatic surgery [33]. Given that diabetes is a significant risk factor for or a complication of PC, it is advisable to incorporate glycemic status or a specified value for glycated hemoglobin in the VTE scale, which may be valuable parameters in PC patients. Currently, limited studies have shown that fasting levels of human pancreatic polypeptide (HPP) are similar in patients with PDAC arising from the head or body/tail [34].

**TABLE 2:** Caprini score [27].

Patient characteristic	Score
Age 41–60 years	1
Planned minor surgery (< 45 min)	1
Major surgery in past 1 month (> 45 min)	1
Visible varicose veins	1
History of inflammatory bowel disease	1
Swollen legs (current)	1
Body mass index ≥ 25 kg/m^2^	1
Myocardial infarction	1
Congestive heart failure	1
Serious infection	1
Existing lung disease	1
On bed rest or restricted mobility for < 72 h	1
Oral contraceptives or hormone replacement therapy	1
Pregnancy or postpartum (< 1 month)	1
History of unexplained stillborn infant, recurrent spontaneous abortion (> 3), premature birth with toxemia, or growth‐restricted infant	1
Age 60–74 years	2
Current or past malignancy (excluding nonmelanoma skin cancers)	2
Planned major surgery (> 45 min)	2
Immobilizing plaster cast in past 1 month	2
Central venous access	2
On bed rest for > 72 h	2
Age 75 years and over	3
History of DVT and/or PE	3
Family history of DVT and/or PE	3
Personal or family history of increased risk of blood clotting	3
Elective hip or knee arthroplasty	5
Hip, pelvis, or leg fracture	5
Serious trauma	5
Spinal cord injury resulting paralysis	5
Stroke	5

**TABLE 3:** Padua score [29].

Patient characteristic	Score
Active cancer^∗^	3
Previous VTE (with the exclusion of superficial vein thrombosis), reduced mobility	3
Reduced mobility^∗∗^	3
Already known thrombophilic condition^∗∗∗^	3
Recent (≤ 1 month) trauma and/or surgery	2
Elderly age (≥ 70 years)	1
Heart and/or respiratory failure	1
Acute myocardial infarction or ischemic stroke	1
Acute infection and/or rheumatologic disorder	1
Body mass index ≥ 30 kg/m^2^	1
Ongoing hormonal treatment	1

^∗^Patients with local or distant metastases and/or in whom chemotherapy or radiotherapy had been performed in the previous 6 months.

^∗∗^Bed rest with bathroom privileges (either due to patient’s limitations or on physicians order) for at least 3 days.

^∗∗∗^Carriage of defects of antithrombin, protein C or S, Factor V Leiden, G20210A prothrombin mutation, antiphospholipid syndrome.

**TABLE 4:** Khorana score (KS) [30].

Patient characteristic	Score
Site of cancer (stomach, pancreas)	2
Site of cancer (lung, gynecological, lymphoma, bladder, testicular, or renal)	1
Prechemotherapy platelet count ≥ 350 × 10^9^/L	1
Prechemotherapy hemoglobin level < 6.2 mmol/L	1
Prechemotherapy leukocyte count > 11 × 10^9^/L	1
Body mass index ≥ 35 kg/m^2^	1

**TABLE 5:** PROTECHT score [31].

Patient characteristic	Score
Site of cancer (pancreatic, gastric, or primary brain cancer)	2
Site of cancer (lung, gynecological, lymphoma, bladder, testicular, or renal)	1
Prechemotherapy platelet count ≥ 350 × 10^9^/L	1
Prechemotherapy hemoglobin level < 6.2 mmol/L	1
Prechemotherapy leukocyte count > 11 × 10^9^/L	1
Body mass index ≥ 35 kg/m^2^	1
Gemcitabine therapy	1
Platinum‐based therapy	1

## 5. Stratifying VTE Risk Factors Based on Tumor Location in the Pancreas

Patients with PDAC at the body/tail often undergo splenectomy during radical surgery, and therefore secondary thrombocythemia is a common postoperative complication frequently present in these patients. The spleen stores circulating erythrocytes and platelets and meanwhile serves as a major site for destruction of these cells [35]. Thrombocytosis secondary to splenectomy is also considered to be caused by the activation of the hematopoietic system in bone marrow as a response to postsurgical trauma. After spleen removal, platelet counts generally increase at 3–9 days and peak around 2 weeks after surgery, usually reaching 300–500 × 10^9^/L in the peripheral blood, and in a few patients even higher [36]. Due to this feedback mechanism, the production of platelets by the bone marrow can be suppressed and platelet counts gradually decline from the first to the second month, slowly returning to a normal range 3–6 months after operations. However, in some patients, thrombocytosis can persist for 12 months or even last for years [37].

Secondary thrombocythemia induced by splenectomy affects VTE formation in PDAC. It has been shown that cancer patients with a high platelet count (> 350 × 10^9^/L) had an increased risk of developing VTE [38]. Activated platelets can release procoagulant factors that promote thrombus formation, while tumor cells can also stimulate the production of tissue factor, a potent initiator of coagulation [39]. In an independent study, the incidence of splenic vein thrombosis (SVT) was observed to be associated with surgeries in patients with PADC originating from the tail, who underwent distal pancreatectomy along with splenectomy [40]. However, in another retrospective study conducted on acute trauma patients who underwent splenectomy, it was revealed that despite an increase in platelets in the postoperative period, secondary thrombocytosis did not serve as an independent risk factor for thrombosis in 1‐year clinical follow‐up [41].

Additionally, in PDAC patients originating from the head of the pancreas often undergo portal vein resection during pancreaticoduodenectomy; this procedure can also disrupt normal hemodynamics, leading to a higher incidence of postoperative PVT [42]. Therefore, monitoring of hemodynamic parameters is recommended in these patients.

To our knowledge, no prospective studies are available to specifically validate the influence of tumors harboring KRAS mutations at different anatomical locations on VTE in pancreatic tumors. However, KRAS mutations are reported to be associated with an increased risk of VTE in patients with NSCLC and metastatic colon cancer, in which KRAS is also frequently mutated [43, 44].

For patients with resectable PDAC, adjuvant chemotherapy is commonly recommended after surgery to improve survival time [45]. Currently, there is no difference in chemotherapeutic regimens used for PDAC derived from head and body/tail origins. The preferred approaches include gemcitabine alone or in combination with the oral fluorouracil analog capecitabine [46]. In recent years, for patients with good PS (ECOG PS score of 0 or 1), a modified mFOLFIRINOX regimen (consisting of oxaliplatin, irinotecan, and 5‐fluorouracil) has been shown to prolong both disease‐free survival (DFS) and overall survival (OS), compared to gemcitabine alone or in combination with gemcitabine and capecitabine [47]. However, FOLFIRINOX‐based vs. gemcitabine‐based regimen reported to have no effect on disease‐free interval as well as the duration of response to first‐line palliative treatment in those with advanced disease in the location of the head or body/tail [48]. Gemcitabine remains the major anticancer regimen in patients with PDAC. As mentioned previously, the PROTECHT score appears to be a valuable tool in stratifying the risk of VTE in patients with secondary thrombocytosis postsplenectomy treated with gemcitabine.

Notably, multiple myelosuppressive effects, typically manifesting as leukopenia, anemia, and thrombocytopenia, are common hematological toxicities induced by gemcitabine. In patients with severe thrombocytopenia, chemotherapy has to be delayed or terminated. It is an interesting question to answer whether the secondary thrombocytosis after PDAC splenectomy is able to compensate for the deceased platelet counts, and subsequently, gemcitabine‐induced thrombocytopenia may be less prevalent in these patients compared to those with PDAC derived from the head. Therefore, patients with splenectomy may be less prone to delay in antitumor drug administration or reduced cycles of chemotherapy compared to those with PDAC originating from the head. Whether the potential variance in the tolerance of adjuvant chemotherapy serves as a risk factor affecting the formation of VTE in head and body/tail PDAC patients differently still requires to be validated.

Taken together, prospective multicenter studies are needed to clarify whether surgical procedures, adjuvant chemotherapy, and other potential risk factors, including tumor location and genetic background, affect the development of VTE in PDAC patients. Furthermore, these studies should aim to develop stratified guidelines for screening patients at risk of VTE.

## 6. Prophylactic Anticoagulation Intervention Affects the Formation of Thromboembolism in Patients With PDAC

**FIGURE 1:**
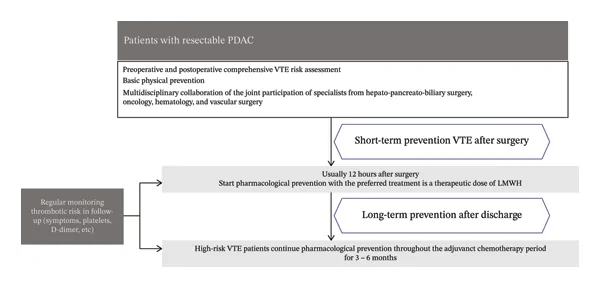
Algorithm for the assessment and management to prevent VTE in patients with resectable PDAC.

Postoperative prophylactic anticoagulation is recommended for patients with various malignancies. Low molecular weight heparin (LMWH) is preferred as the first‐line anticoagulant at the highest feasible dose. LMWH is commonly prescribed approximately 12 h after surgery and continued for 10–14 days [49]. Additionally, non–vitamin K antagonist oral anticoagulants (NOACs), including direct thrombin inhibitors (e.g., dabigatran) and factor Xa inhibitors (rivaroxaban, apixaban, and edoxaban), have been tested for their efficacy and safety in the prevention of VTE after surgery. These drugs have advantages including easy administration, rapid onset of action, and a short half‐life. For instance, the CASSINI study was a prospective, large‐scale, randomized, controlled, Phase III clinical trial that aimed to assess the efficacy and safety of rivaroxaban in preventing VTE in cancer patients undergoing chemotherapy who had a Khorana score ≥ 2 [50]. Among all enrolled patients, 32.5% of patients developed PC [50]. In this trial, all participants were randomly allocated to either the rivaroxaban group (receiving 10 mg/day) or the placebo group after the initiation of chemotherapy for 180 days. The data showed a reduced incidence of VTE events in the rivaroxaban group compared to the placebo group, without a significant increase in the risk of hemorrhage and VTE‐related mortality between the two groups [50]. Prior to the trial, routine ultrasound screening was conducted and revealed that 5% of enrolled patients already exhibited thrombosis [50]. A visual algorithm is provided to illustrate the assessment and management to prevent VTE in patients with resectable PDAC (Figure 1). The American Society of Clinical Oncology (ASCO) guidelines still encourage the use of risk assessment models to determine the risk of VTE, such as the Khorana risk score = 0 is regarded low risk and ≥ 2 is regarded high risk.

However, in this study, the occurrence of VTE and VTE‐related mortality between head and body/tail PDAC patients was not distinguished in the subsequent prophylactic anticoagulation usage. PVT is more common in head PDAC, which is characterized by the tumor’s proximity to the portal vein and the superior mesenteric vein. In contrast, body and tail PCs are located near the splenic vein, making SVT more prevalent. This divergence in anatomical positioning not only influences the type of thrombosis that may occur but also complicates the clinical management of these patients, as the therapeutic approach may differ significantly from those with head PC. The duration of anticoagulation therapy can vary significantly based on the thrombus location and the patient’s clinical status. For example, patients with PVT may require prolonged anticoagulation, often extending beyond 6 months, particularly if they have underlying prothrombotic conditions or if the thrombus is extensive [51]. In contrast, SVT may necessitate a shorter duration of therapy, especially if it is asymptomatic and does not pose a risk for significant complications. Additionally, SVT presents unique challenges in anticoagulation management. The survey revealed that SVT is less commonly treated with anticoagulation, with 86% of pancreatologists indicating a lower preference for initiating therapy in this location [51]. The rationale is that SVT may not present the same immediate risks as PVT. Therefore, management often involves observation unless significant complications or symptoms arise.

Understanding these differences is crucial for an individualized approach to anticoagulation therapy. Such an approach balances the risks of thrombosis recurrence against the potential for bleeding complications. The management of PC‐related VTE requires multidisciplinary collaboration. By establishing standardized monitoring procedures and prophylactic anticoagulation intervention guidelines, clinicians can ensure that patients with PDAC achieve enhanced quality of life, reduced thrombotic risks, and optimized clinical outcomes.

## 7. Limitations

This mini‐review article has several limitations. First, it excludes a substantial amount of high‐quality literature published earlier than 2010 and in other languages. Additionally, the quality of the included reviews and original articles was not formally evaluated. Finally, many studies were included through thorough searches of the PubMed database; however, some articles in other major electronic databases were not included in this review.

## 8. Conclusion

In summary, PDAC has a low 5‐year survival rate and a high incidence of VTE, which significantly worsens prognosis. Distinct surgical approaches for PDAC based on the tumor’s anatomical location lead to varying postoperative complications. For example, in PDAC arising from the body and tail regions, splenectomy often leads to secondary thrombocythemia—a condition characterized by an elevated platelet count—which may increase the risk of thrombosis. Therefore, refined risk assessment tools are essential for identifying patients at high risk of VTE occurrence in PDAC patients with tumors located in different regions of the pancreas and facilitating the implementation of prophylactic strategies.

## Funding

This work was supported by Science and Technology Bureau of Jiaxing (grant no.: 2025CGZ054) and Starlit South Lake Leading Elite Program (grant no.: 2023A400006).

## Ethics Statement

The authors have nothing to report.

## Conflicts of Interest

The authors declare no conflicts of interest.

## Data Availability

The data that support the findings of this study are available from the corresponding author upon reasonable request.
